# Curcumin reduces mitomycin C resistance in breast cancer stem cells by regulating Bcl-2 family-mediated apoptosis

**DOI:** 10.1186/s12935-017-0453-3

**Published:** 2017-09-26

**Authors:** Qian-Mei Zhou, Yang Sun, Yi-Yu Lu, Hui Zhang, Qi-Long Chen, Shi-Bing Su

**Affiliations:** Research Center for Traditional Chinese Medicine Complexity System, Shanghai University of Traditional Chinese Medicine, Shanghai, 201203 China

**Keywords:** Curcumin, Resistance, Breast cancer stem cells, Bcl-2 family, Apoptosis

## Abstract

**Background:**

Curcumin, a natural compound derived from the turmeric rhizome *Curcuma longa* Linn, has anticancer and chemoresistance reduction biological activities. We evaluated the efficacy of curcumin in sensitizing chemotherapy drugs through regulation of Bcl-2-mediated apoptosis in breast cancer stem-like cells (BCSCs).

**Methods:**

Cell survival was measured using MTT assay. Apoptosis-related proteins were observed using western blot analysis. Apoptosis was detected with flow cytometric analysis and by Hoechst 33258 staining. The mitochondrial membrane potential was observed with flow cytometric analysis.

**Results:**

The ability of BCSCs to propagate decreased gradually along the passages and was completely lost at the fifth passage [0.1 μmol/L mitomycin C (MMC) with 5 μmol/L curcumin in MCF-7 and 0.5 μmol/L MMC with 5 μmol/L curcumin in MDA-MB-231 cells]. Curcumin combined with MMC treatment significantly decreased the levels of antiapoptotic Bcl-2 and Bcl-w expression, increased the levels of proapoptotic Bax, Bak, Bad, Bik, and Bim expression, and activated caspase-3 and caspase-9 in MCF-7 BCSCs. In the presence of Bcl-2 siRNA, the apoptosis rate increased by 15% in cells treated with curcumin and MMC. The mitochondrial membrane potential decreased by approximately 20% in MCF-7 BCSCs undergoing the combination treatment of curcumin and MMC. The combination-induced decrease in Bcl-2 was regulated by the presence of the Wnt-specific inhibitor PFK115-584 and PI3k inhibitor LY294002.

**Conclusions:**

Our study indicates that curcumin might represent a novel therapeutic agent for treating breast cancer chemoresistance induced by MMC.

## Introduction

Breast cancer prevalence is growing worldwide. More than 1.3 million women worldwide are diagnosed every year [1]. Chemotherapy, surgery, and radiation are available for treatment of the primary tumor in breast cancer. However, chemoresistance remains a major obstacle to the treatment of breast cancer, and leads to poor prognoses [2]. Resistance occurs not only at the beginning of chemotherapy but also following successful chemotherapy. The chemoresistance procedure and the molecular mechanisms of drug resistance provide targets for chemotherapy drugs [3].

Apoptosis regulator Bcl-2 is a family of evolutionarily related proteins. These proteins govern mitochondrial outer membrane permeabilization and can be either proapoptotic (Bax, Bad, and Bak) or antiapoptotic (Bcl-2 proper, Bcl-XL, Mcl, and Bcl-w). Bax and Bak are effector proteins [4]. The activity of Bax and Bak is constrained by the prosurvival Bcl-2 proteins that prevent their homomultimerization. Proteins that stimulate or inhibit apoptosis interact with each other and determine the death or survival of cells [5]. In many tumors, overexpression of antiapoptotic Bcl-2 proteins contributes to apoptosis resistance. Bcl-2 and Bcl-XL have been identified as two key inhibitors of various apoptotic stimuli. Bax and Bak enable the effector function for apoptotic stimuli. Bid and Bim can activate Bax and Bak through direct interaction. Bad activates Bax and Bak from their proapoptosis counterparts [6].

The overexpression of antiapoptotic Bcl-2 plays a critical role in conventional anticancer therapeutic resistance [7]. There is an inverse correlation between Bcl-2 expression and chemosensitivity to chemotherapy drugs such as adriamycin and 5-fluorouracil (5-FU) in breast cancer cells [8]. Bcl-2 and Bcl-XL were determined to be crucial for the induction of paclitaxel resistance in human hepatocellular carcinoma cells [9]. The antiapoptotic protein Bcl-2 is localized in the nucleus, mitochondria, and estrogen receptor (ER). Apoptosis was associated with dissipation of the mitochondrial membrane potential. Apoptosis in T cells of susceptible mice was associated with altered induction of Bcl-2/Bax and loss of mitochondrial transmembrane potential [10].

Bcl-2 inhibitors overcome resistance in patient-derived glioblastoma stem-like cells [11], and they may overcome resistance to 5-FU-based chemotherapy and consequently improve outcomes in patients with colorectal cancer [12]. Nicotine activates downstream signaling of Bcl-2 inhibitors, interfering with the ubiquitination process and preventing Bcl-2 from being degraded in lung cancer cells, resulting in increased chemoresistance [13]. Upregulation of the Bcl-2 family was implicated in intrinsic gemcitabine resistance in pancreatic and lung cancers. These findings have suggested that increased levels of Bcl-2 family proteins play an important role in the development of chemotherapy resistance. Therefore, research into the role of Bcl-2 family proteins can help us understand the development of tumor chemotherapy resistance.

Curcumin, the principal polyphenolic curcuminoid, is extracted from the turmeric rhizome *Curcuma longa* Linn and has been extensively researched for its biological properties, which include anti-inflammatory, antioxidant, anti-infection, and anticancer activities. Curcumin has a potent antitumor effect and is safe for consumption [14–16]. Curcumin inhibits cancer cell proliferation [17] and migration [18], induces apoptosis [19], and sensitizes cancer cells to chemotherapy drugs [20] through regulation of Bcl-2 family proteins. Moreover, curcumin overcomes multidrug resistance in various cancers [21, 22].

We previously showed that curcumin improved the antitumor effects of mitomycin C (MMC) on breast cancer cells [14–16]. However, the mechanism associated with curcumin-mediated drug sensitization is unknown. Curcumin can inhibit the growth of cancer stem-initiating cells [23–25]. Thus, we hypothesized that curcumin-mediated chemosensitization is due to its ability to target cancer stem-like cells through Bcl-2 family-mediated apoptosis. In this study, we showed that curcumin sensitized breast cancer stem-like cells (BCSCs) to MMC through apoptosis by regulating the imbalance of Bcl-2 family proteins, which decreased mitochondrial transmembrane potential. Moreover, the combination treatment inhibited the expression of Bcl-2 via the Wnt and PI3k pathways.

## Materials and methods

### Materials

MMC was purchased from ICN Company (USA), dissolved in physiological saline as a 1 mmol/L stock solution, and stored at 4 °C away from light. Curcumin, with a purity of more than 98%, was obtained from the National Institute for the Control of Pharmaceutical and Biological Products (China). Curcumin was dissolved in dimethyl sulfoxide (DMSO) as a 40 mmol/L solution. PFK115-584, cyclopamine, LY294002, SP600125, PD98059, SB203580, and GSIs were obtained from Biomol (Philadelphia, PA, USA). The antibodies against Bcl-2, Bcl-XL, Bcl-w, Bax, Bak, Bid, Bad, Bim, mcl-1, p53, caspase-3, caspase-8, caspase-9, β-catenin, GSK-3β, TCF, LEF, Akt, p-Akt, NF-κB (p65), and IκBɑ were obtained from Cell Signaling Inc. (Boston, MA, USA).

### Cell culture, mammosphere-forming assay, and self-renewal analysis

The human breast cancer cell lines MDA-MB-231 and MCF-7 were purchased from the American Type Culture Collection (Rockville, MD, USA) and cultured in DMEM (Gibco, Scotland, UK) supplemented with 10% heat-inactivated fetal bovine serum (Gibco, Scotland, UK) at 37 °C in a humidified incubator supplied with 5% CO_2_. Mammospheres were generated by seeding MDA-MB-231 and MCF-7 cells at 10^3^ cells/cm^2^ in six-well ultralow attachment plates in mammosphere medium (F-12/DMEM containing 5 mg/mL insulin, 2% B27, 10 ng/mL basic fibroblast growth factor, and 20 ng/mL human recombinant epidermal growth factor).

Unsorted single MDA-MB-231 and MCF-7 cells were cultured in suspension in serum-free media with curcumin, MMC, or curcumin together with MMC, and primary spheres were collected, dissociated, and resuspended in mammosphere medium to form secondary spheres. The secondary spheres were counted after 7 days, dissociated again, and recultured to form tertiary spheres.

### MTT assays

Cancer stem-like cells were plated onto 96-well plates in stem cell culture medium containing various concentrations of drugs. Cell viability was assessed using the MTT assay as previously described (Promega, Madison, WI). Cytotoxicity was expressed as the percentage of surviving cells (total number of untreated cells).

### Measurement of mitochondrial membrane potential

The effect of PEITC treatment on mitochondrial membrane potential was measured using the potential-sensitive dye JC-1 (5, 5′, 6, 6′-tetrachloro-1, 1′, 3, 3′-tetraethyl benzimidazolyl carbocyanine iodide) according to the manufacturer’s instructions. Stock solution of JC-1 (1 mg/mL) (Becton–Dickinson, CA, USA) was prepared in DMSO and freshly diluted with the assay buffer supplied by the manufacturer. MCF-7 BCSCs (4 × 10^5^) were plated in 25 cm^2^ culture flasks, allowed to attach overnight, exposed to desired concentrations of curcumin and MMC for a specified time period, and collected through trypsinization. The cells were incubated in a medium containing JC-1 (10 μg/mL) for 15 min at 37 °C. Cells were washed and resuspended in 0.5 mL of assay buffer and the fluorescence was measured using a fluorescence-activated cell sorter (FACS) (Becton–Dickinson, CA, USA).

### siRNA transfection

Transfection was performed with Lipofectamine 2000 (Invitrogen, California, USA) by following the manufacturer’s instructions. siRNA transfection was performed 24 h before curcumin and MMC treatment. siRNA duplexes, including Bcl-2 siRNA (sc-29214) and control scrambled siRNA (sc-37007), were obtained from Santa Cruz Biotechnology.

### Flow cytometry analysis of BCSCs

Flow cytometric analysis was performed on MCF-7 cells (10^6^/mL) cultured in six-well plates. Cells reached 70–80% confluence after being seeded for 12 h. Cells were treated with MMC at 0.1 μmol/L, curcumin at 5 μmol/L, or MMC at 0.1 μmol/L with curcumin at 5 μmol/L, or Bcl-2 siRNA alone or together for 7 days. Cells were subjected to an annexin V-PI dual staining assay according to the manufacturer’s protocol. Stained cells were analyzed using the FACS, and the percentage of apoptotic cells in the population was determined using ModFit LT 3.0 software (Becton–Dickinson, CA, USA).

### Hoechst 33258 staining

MCF-7 BCSCs in the logarithmic growth phase were seeded in 96-well plates at a density of 1 × 10^4^/mL. Different treatment groups and the control group were cultured for 7 days. Cells were fixed with 3.7% paraformaldehyde for 30 min at room temperature, and then washed and stained with 167 μmol/L Hoechst 33258 at 37 °C for 30 min. Cells were observed under a fluorescence microscope (Olympus, Japan; Nikon, Tokyo, Japan) equipped with a UV filter. The images were recorded on a computer with a digital camera attached to the microscope, and the images were processed by the computer. The Hoechst reagent was absorbed by the nuclei of the cells, with apoptotic cells exhibiting a bright blue fluorescence.

### Western blot analysis

Cells were directly lysed in lysis buffer containing 2 mol/L sodium chloride, 10% NP-40, 10% SDS, 1 mol/L Tris–Cl, 1 g/L phenyl-methylsulfonyl fluoride, 0.1 g/L aprotinin, and 0.01 g/L leupeptin. The cell lysates were subjected to SDS-PAGE and then blotted onto PVDF membranes. After the membranes were blocked with BSA for 1 h, the protein expression was detected using primary antibodies (1:1000) and secondary antibodies (1:800) conjugated with horseradish peroxidase and enhanced chemiluminescence reagents (Pharmacia, Buckinghamshire, UK). Western blots were quantitatively analyzed using Alpha Ease FC (FluorChem FC2) software. The density ratio of proteins to GAPDH as the spot density was calculated using the analytical tools.

### Statistical analysis

Statistical differences were identified using two-tailed Student t tests. Data are presented as the mean ± SD. P < 0.05 was considered to be significant.

## Results

### Curcumin significantly enhances the capability of MMC to reduce the self-renewal of stem-like breast cancer cells

In a previous study, we found that, with 5 μmol/L curcumin, MMC reduced mammosphere-forming efficiency (MSFE) by 50% at 0.1 μmol/L in MCF-7 cells and 0.5 μmol/L in MDA-MB-231 cells, representing 5- and 15-fold decreases in the concentration required to achieve such a reduction, respectively (data not shown). The ability of mammospheres to be serially passaged at clonal density is an indirect marker of stem cell self-renewal [26].

**Fig. 1:**
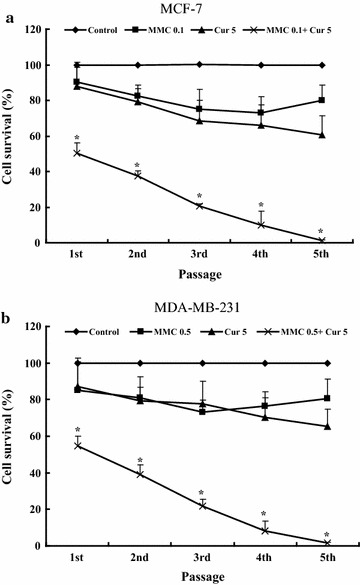
Curcumin and MMC alone or together inhibited self-renewal of MCF-7 and MDA-MB-231 BCSCs. The self-renewal capacity of BCSCs was observed with **a** curcumin at 5 μmol/L and MMC at 0.1 μmol/L alone or together in MCF-7 cells and **b** curcumin at 5 μmol/L and MMC at 0.5 μmol/L alone or together in MDA-MB-231 cells for 7 days. Data are presented as the mean ± SD (n = 3). *P < 0.05 vs. control in different passages

We investigated the effects of MMC on the self-renewal of MDA-MB-231 and MCF-7 cells with or without curcumin. On the basis of the formation of mammosphere structures, BCSCs derived from MCF-7 and MDA-MB-231 cells were able to propagate in media containing 0.1 μmol/L MMC with 5 μmol/L curcumin and 0.5 μmol/L MMC with 5 μmol/L curcumin, respectively. However, the ability of BCSCs to propagate decreased gradually along the passages and was completely lost at the fifth passage (Fig. 1). Since neither MMC nor curcumin alone significantly reduced the propagation of BCSCs along the passages, these results suggest that the combination of MMC and curcumin can effectively block the self-renewal of BCSCs.

### Curcumin enhances the efficacy of MMC to induce BCSC death by regulating Bcl-2 family protein expression

**Fig. 2:**
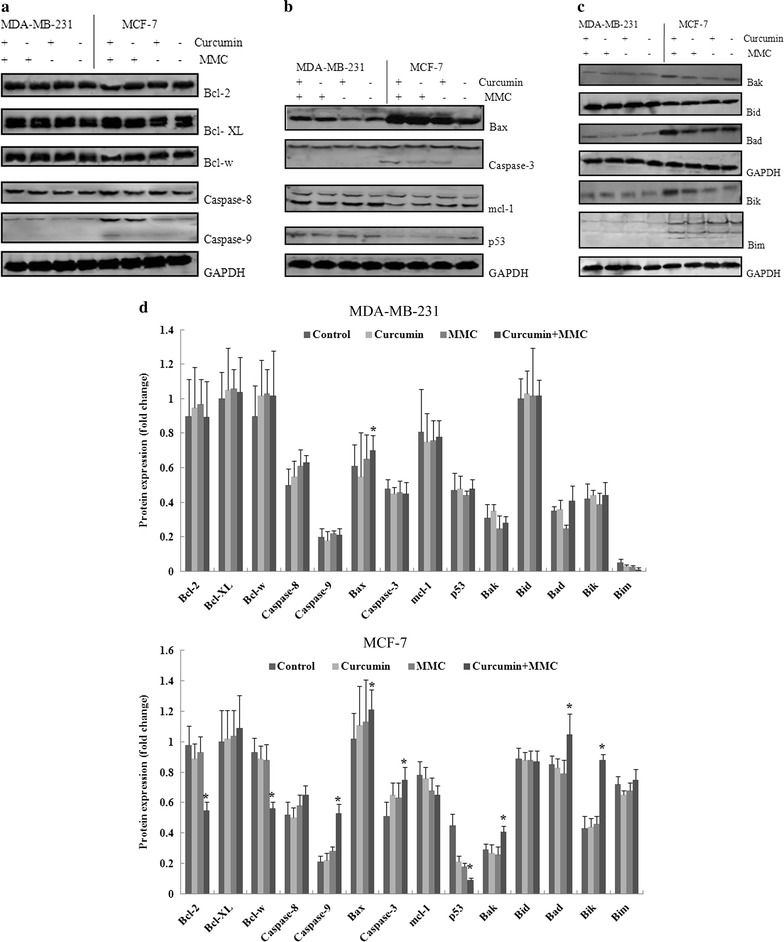
Effect of the combination treatment of curcumin and MMC on the expression of Bcl-2 family proteins. Western blot analysis of **a** Bcl-2, Bcl-XL, Bcl-w, caspase-8, and caspase-9; **b** Bax, caspase-3, mcl-1, and p53; and **c** Bak, Bid, Bad, Bik, and Bim upon treatment with curcumin and MMC alone or together in MDA-MB-231 and MCF-7 BCSCs. Cells were incubated with 5 μmol/L curcumin and 0.5 μmol/L MMC in MDA-MB-231 cells and 0.1 μmol/L MMC in MCF-7 cells and their combinations over 7 days. **d** The density ratio of proteins to GAPDH is shown as relative expression. Values are expressed as the mean ± SD (n = 3). Experiments were repeated with similar results. *P < 0.05 vs. untreated control

A hallmark of cancer stem cells is the enhanced expression of Bcl-2 family proteins that can protect them from damage induced by cytotoxic agents. The observation that curcumin enhances the ability of MMC to induce BCSC death led to the hypothesis that curcumin exerts its effect by modulating Bcl-2 family protein expression in BCSCs. To test this hypothesis, we analyzed the effect of curcumin on the levels of Bcl-2 family proteins in BCSCs. Western blot analyses showed that curcumin combined with MMC treatment blocked the expression of Bcl-2 and Bcl-w in MCF-7 BCSCs, compared with untreated cells, but not in MDA-MB-231 BCSCs (Fig. 2). In addition, curcumin combined with MMC increased the levels of Bax, Bak, Bad, Bik, and Bim in MCF-7 BCSCs. Caspase-3 and caspase-9 were also activated by curcumin combined with MMC. These results suggest that curcumin increases MMC-mediated cell death in BCSCs by regulating Bcl-2 family protein expression.

### Curcumin combined with MMC induces apoptosis through Bcl-2

We found that curcumin combined with MMC increased proapoptotic protein expression (Bax, Bak, Bad, Bik, and Bim) and decreased antiapoptotic protein expression (Bcl-2 and Bcl-w) in MCF-7 cells (Fig. 2). The observation that curcumin enhances the ability of MMC to induce BCSC apoptosis led to the hypothesis that curcumin exerted its effect by modulating Bcl-2 expression in BCSCs. To test this hypothesis, we treated MCF-7 BCSCs with Bcl-2 siRNA. We detected the apoptosis of MCF-7 BCSCs through flow cytometric and Hoechst 33258 staining analyses. In the presence of Bcl-2 siRNA, the apoptosis rate increased by 15% in cells treated with curcumin combined with MMC compared with treatment without Bcl-2 siRNA. These results suggest that curcumin increases MMC-mediated apoptosis in BCSCs by blocking Bcl-2 expression.

### Curcumin combined with MMC disrupts mitochondrial membrane potential

Activation of caspase-9 in response to various stimuli is often associated with disruption of the mitochondrial membrane potential [22]. We have shown that curcumin with MMC-induced apoptosis in MCF-7 BCSCs is associated with the activation of caspase-3 and caspase-9. In this study, we also determined whether curcumin with MMC-induced apoptosis was associated with disruption of the mitochondrial membrane potential.

The effect of curcumin with MMC treatment on mitochondrial membrane potential was determined through flow cytometry following staining with the potential-sensitive dye JC-1. Representative histograms are shown in Fig. 4. The mitochondrial membrane potential decreased by approximately 20% in MCF-7 BCSCs treated with the combination treatment of curcumin and MMC, compared with curcumin and MMC alone.

### Curcumin combined with MMC inhibits Bcl-2 expression via the Wnt and PI3k pathways

To investigate which pathways regulate Bcl-2 expression by curcumin combined with MMC, we treated MCF-7 BCSCs with specific inhibitors of Wnt, Hedgehog, PI3k, NF-κB, JNK, ERK, MAPK, and Notch. The results suggested that these inhibitors inhibited their respective pathways (Fig. 5a).

MCF-7 BCSCs were pretreated for 2 h with or without these inhibitors at 10 μmol/L followed by exposure to 5 μmol/L curcumin in combination with 0.1 μmol/L MMC for 7 days. As shown in Fig. 5b, the combination-induced decrease in Bcl-2 was reversed to the basal level in the presence of the Wnt-specific inhibitor PFK115-584. The PI3k inhibitor LY294002 also inhibited Bcl-2 expression following the combination treatment (Fig. 5c). However, under similar experimental conditions, the expression of Bcl-2 was unaffected by other inhibitors, suggesting that Bcl-2 is not likely to be involved in Hedgehog-, NF-κB-, JNK-, ERK-, MAPK-, or Notch-mediated apoptosis that is induced by the combination treatment of curcumin and MMC (Fig. 6a, b).

### Curcumin combined with MMC inhibits cell survival and induces apoptosis through Wnt and PI3k pathways

To observe the effect of curcumin combined with MMC on the Wnt and PI3k pathways, the associated proteins were detected. MCF-7 BCSCs were pretreated for 2 h with or without PFK115-584 and LY294002 at 10 μmol/L followed by exposure to 5 μmol/L curcumin in combination with 0.1 μmol/L MMC for 7 days. Figure 7a, b shows that the combination treatment decreased the expression of β-catenin, TCF, p-Akt, and NF-κB (p65) and increased the levels of GSK-3β and IκBɑ. However, the expression of LEF and Akt was unaffected. Therefore, the Wnt and PI3k pathways were regulated by the combination treatment of curcumin and MMC.

The inhibition of Bcl-2 was regulated through the Wnt and PI3k pathways. To investigate whether curcumin enhances the ability of MMC to induce BCSC death, we hypothesized that curcumin combined with MMC exerts its effect by modulating the Wnt and PI3k pathways in BCSCs. MCF-7 BCSCs were pretreated for 2 h with or without 10 μmol/L PFK115-584 or LY294002 followed by treatment with 5 μmol/L curcumin, 0.1 μmol/L MMC, or both for 3, 5, or 7 days. Cell viability and apoptosis were determined using the MTT assay and flow cytometry analysis, respectively. After treatment with the combination of curcumin and MMC, curcumin and MMC with PFK115-584, and curcumin and MMC with LY294002, cell growth inhibition was approximately 18, 10, and 14% at 3 days, 32, 18, and 49% at 5 days, and 48, 20, and 62% at 7 days, respectively (Fig. 7c). Significant differences were observed between curcumin with MMC, curcumin with MMC and PFK115-584, and curcumin with MMC and LY294002 at 5 and 7 days (P < 0.05). The apoptosis rate was approximately 36, 5, and 54% after 7 days (P < 0.05) compared with curcumin with MMC (Fig. 7d). These results suggest that the inhibitory effect and apoptosis were reversed by pretreatment with PFK115-584. However, LY294002 enhanced these effects with the combination of curcumin and MMC. Therefore, the cell growth inhibition and apoptosis caused by the combined treatments are dependent on the Wnt and PI3k pathways in different manners.

## Discussion

MMC is an anticancer drug that is used clinically for patients with various types of cancer. However, tumor cell resistance to this agent remains the main obstacle in successful cancer therapy. Cancer stem cells represent a small number of pluripotent and self-renewing cells within a tumor that are resistant to conventional chemotherapy and are responsible for tumor initiation and maintenance [27]. Moreover, the clinical dosage of MMC causes severe renal toxicity. These side effects have limited the use of MMC in cancer chemotherapy [28].

Research has addressed the chemotherapeutic potential of curcumin (diferuloylmethane). Curcumin, a nontoxic plant-derived polyphenol [29] and natural plant phenolic food additive, is an active component of the perennial herb *C. longa* (commonly known as turmeric) [30]. It has been shown to exhibit antitumorigenic effects in various types of cancer. For instance, curcumin reverses cisplatin resistance in cervical cancer cells [31] and reduces multidrug resistance in human colon cancer [32]. The exact mechanisms that mediate the chemotherapy resistance of Bcl-2 family proteins resulting from the combined treatment of curcumin and MMC have not been fully explained.

In this study, treatment of MCF-7 and MDA-MB-231 BCSCs with either curcumin or MMC alone resulted in cell growth inhibition in a dose-dependent manner (data not shown), which is consistent with previously published data [33]. Moreover, the combination treatment of 5 μmol/L curcumin with 0.1 μmol/L MMC in MCF-7 BCSCs and 5 μmol/L curcumin with 0.5 μmol/L MMC in MDA-MB-231 BCSCs effectively blocked the self-renewal of BCSCs. The ability of BCSCs to proliferate decreased across the passages and was completely lost at the fifth passage (Fig. 1). These results suggest that curcumin in combination with MMC enhances inhibition of BCSC self-renewal more significantly than MMC alone does. The mechanisms through which curcumin reduces drug resistance involve the activity of ATP-binding cassette transporters [34], ectopic expression of Hsp70 [35], and modulatory effects on multidrug resistance-associated protein 5 [36]. We investigated the role of apoptosis-related proteins including Bcl-2 family and caspase family proteins in BCSCs by combining curcumin and MMC treatment.

**Fig. 3:**
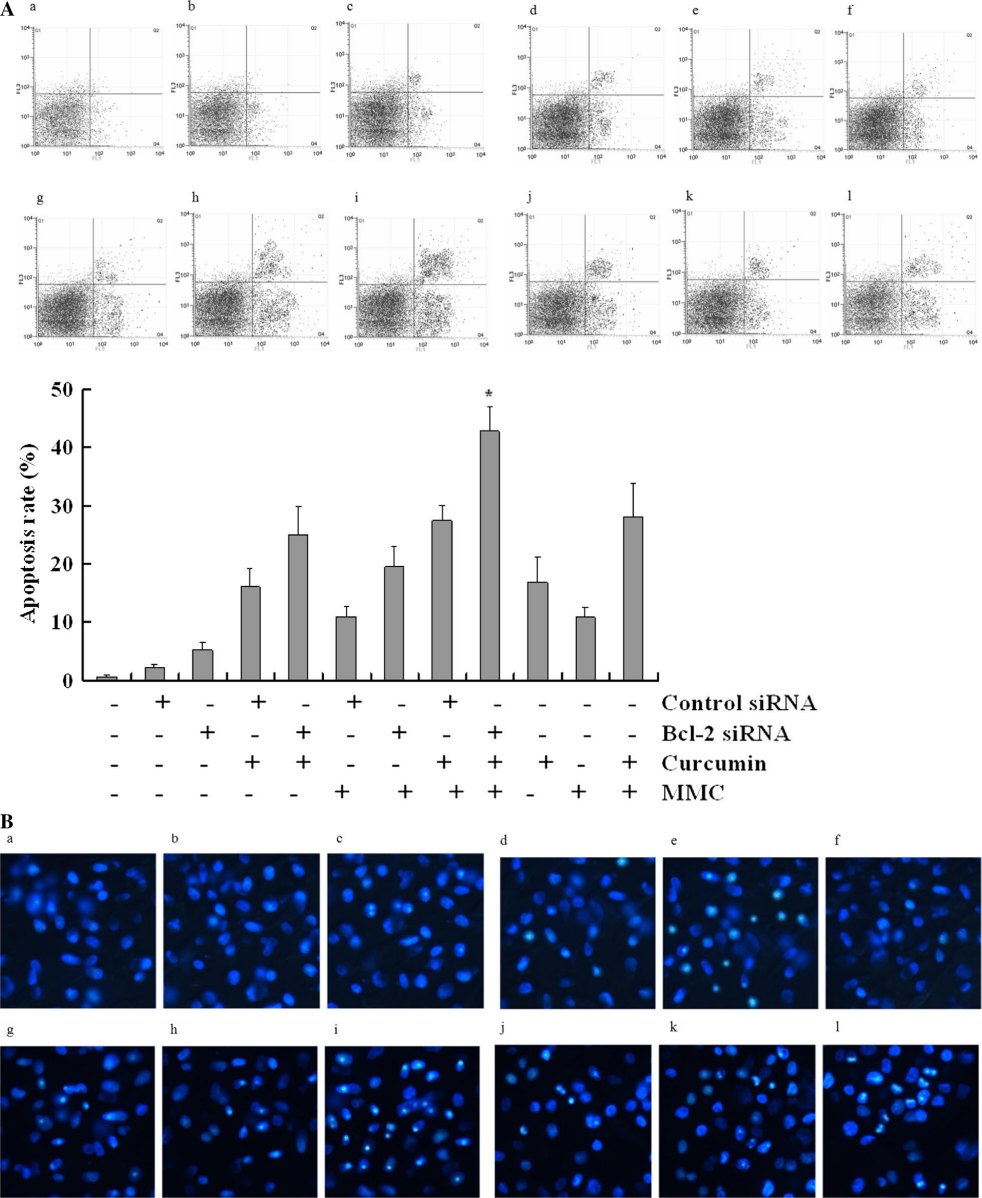
Effect of Bcl-2 siRNA on apoptosis with the combination of curcumin with MMC in MCF-7 BCSCs. Apoptosis of MCF-7 BCSCs following treatment with curcumin at 5 μmol/L and MMC at 0.1 μmol/L for 7 days was analyzed after Bcl-2 or control siRNA treatment. **A** Cells were subsequently subjected to Annexin V-PI staining and flow cytometric analysis. **B** Cell morphology was observed through Hoechst 33258 staining and observed under a fluorescence microscope (× 200). Data are expressed as the mean ± SD (n = 3). *P < 0.05 vs. the combination treatment of curcumin and MMC. **a** Untreated control; **b** control siRNA; **c** Bcl-2 siRNA; **d** control siRNA and curcumin; **e** Bcl-2 siRNA and curcumin; **f** control siRNA and MMC; **g** Bcl-2 siRNA and MMC; **h** control siRNA, curcumin, and MMC; **i** Bcl-2 siRNA, curcumin, and MMC; **j** curcumin; **k** MMC; **l** curcumin and MMC

Bcl-2 is an antiapoptotic member in the initiation of cell death. Proteins that stimulate or inhibit apoptosis determine cell death or survival. Overexpression of antiapoptotic Bcl-2 occurs in many cancer cells and prevents cell death induced by nearly all anticancer drugs and radiation. Our findings demonstrated that the expression of Bcl-2 was significantly increased in BCSCs, which is consistent with a previous study [37]. Many of the components of Bcl-2 family proteins are currently being explored as markers for tumorigenesis and as potential targets for treatment [38]. Our results showed that curcumin combined with MMC treatment induced apoptosis that was inhibited by Bcl-2 siRNA (Fig. 3), blocked the expression of the antiapoptotic members Bcl-2 and Bcl-w, and increased the expression levels of the proapoptotic members Bax, Bak, Bad, Bik, and Bim in MCF-7 BCSCs (Fig. 2). They also indicated that curcumin is able to sensitize MMC-treated BCSCs to apoptosis induction by regulating the imbalance of Bcl-2 family proteins.

The combination of curcumin and MMC significantly reduce the MDA-MB-231 and MCF-7. It has been reported that curcumin induced apoptosis in MDA-MB-231 cells through other pathways [39–41]. Moreover, MDA-MB-231 cells are more metastatic than MCF-7. In this study, we selected MCF-7 cells for apoptosis and related pathways.

**Fig. 4:**
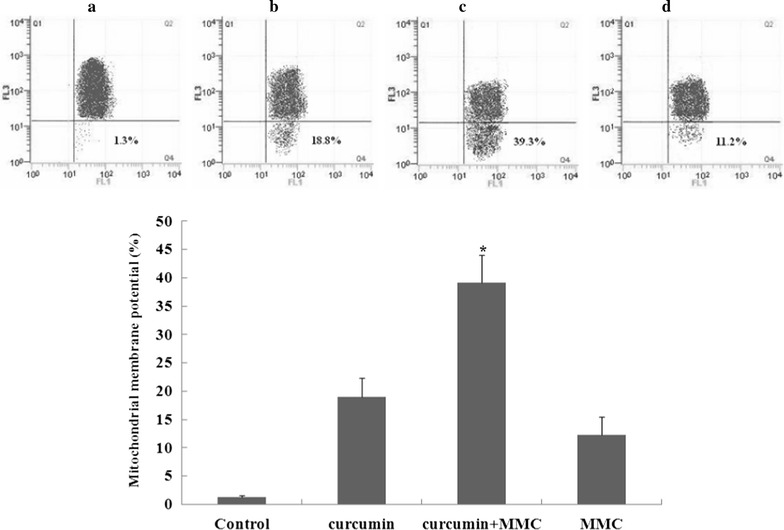
Effect of mitochondrial membrane potential on the combination of curcumin and MMC in MCF-7 BCSCs. The mitochondrial membrane potential of MCF-7 BCSCs following treatment with curcumin at 5 μmol/L and MMC at 0.1 μmol/L for 7 days was analyzed through flow cytometry. Representative histograms are shown. **a** Untreated control; **b** curcumin alone; **c** curcumin and MMC; **d** MMC. Data are expressed as the mean ± SD (n = 3). *P < 0.05 vs. curcumin or MMC treatment alone

The proapoptotic and antiapoptotic members of the Bcl-2 family are integrated with survival signals and coupled to the activation of caspases [42]. Stimulation of apoptotic pathways could lead to the activation of caspases. Caspases are classified as initiators (caspase-8 and -9) and effectors (caspase-3, -6, and -7). Caspase-8 is involved in the receptor-dependent apoptotic pathway, whereas caspase-9 is a mitochondria-dependent apoptosis caspase [43]. The expectation was that in MCF-7 BCSCs, curcumin with MMC would activate caspase-3 and caspase-9 (Fig. 2). The results suggested that curcumin increases MMC-mediated cell death in BCSCs by regulating Bcl-2 family protein expression via the mitochondria-dependent pathway. Furthermore, curcumin-treated BCSCs exhibited the typical features of apoptotic cell death, including shrinkage, transient phosphatidylserine exposure, increased membrane permeability, and a decrease in mitochondrial membrane potential [44]. In this study, the combination treatment of curcumin and MMC also reduced mitochondrial membrane potential in MCF-7 BCSCs by approximately 20% compared with the treatment with curcumin or MMC separately (Fig. 4). These results suggested that curcumin increases MMC-disrupted mitochondrial membrane potential in MCF-7 BCSCs.

The Wnt/β-catenin signaling pathway drives stem cell self-renewal and is involved in various types of cancer. Aberrant activation of the Wnt signaling pathway in normal stem cells can promote their transformation into cancer stem cells [45]. Thus, drugs that target the Wnt/β-catenin pathway may effectively eliminate cancer stem cells [46]. The apoptosis of cisplatin-resistant lung cancer cells is induced through the Wnt pathway [47]. Curcumin promotes the apoptosis of human endometrial cancer cells via the Wnt signal pathway [48]. Previous studies have also demonstrated that PI3k is involved in apoptosis in cancer stem cells. Evidence indicates that the inhibition of the PI3K/Akt/mTOR pathway primes cancer cells for mitochondrial apoptosis by tipping the balance toward antiapoptotic Bcl-2 proteins, resulting in increased mitochondrial outer membrane permeabilization. Thus, mitochondrial apoptotic events play an important role in PI3k inhibitor-mediated sensitization for apoptosis [49].

**Fig. 5:**
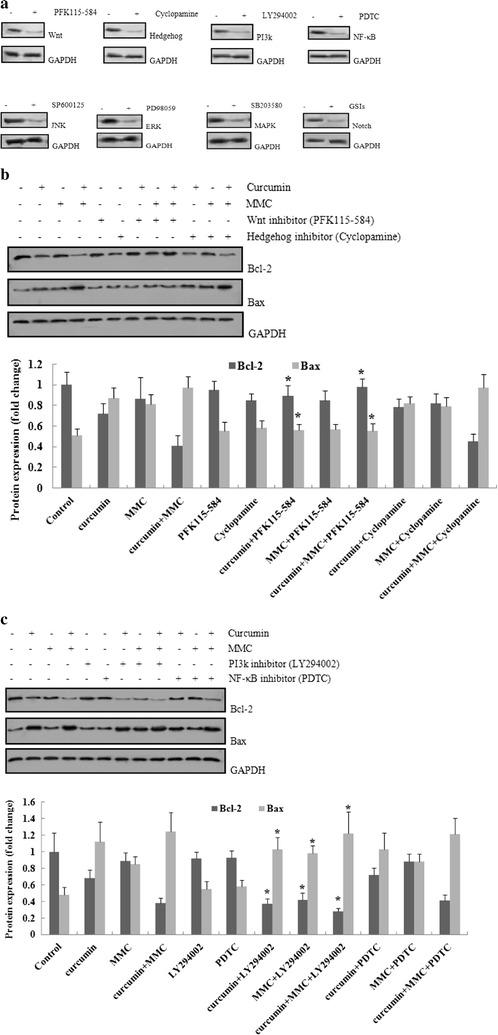
Effect of specific inhibitors of Wnt, Hedgehog, PI3k, NF-κB, JNK, ERK, MAPK, and Notch on Bcl-2 expression that was decreased by the combination treatment of curcumin and MMC. **a** MCF-7 BCSCs were treated with specific inhibitors of Wnt, Hedgehog, PI3k, NF-κB, JNK, ERK, MAPK, and Notch for 12 h. The expression of Wnt, Hedgehog, PI3k, NF-κB, JNK, ERK, MAPK, and Notch was observed through western blot analysis. MCF-7BCSCs were plated and preincubated for 2 h in the presence of **b** PFK115-584 and cyclopamine; **c** LY294002 and PDTC. Cells were then treated with 5 μmol/L curcumin and 0.1 μmol/L MMC alone or together for 7 days. Western blot analysis was then performed. The density ratio of proteins to GAPDH is shown as relative expression. Values are expressed as the mean ± SD (n = 3). Experiments were repeated with similar results. *P < 0.05 vs. specific inhibitors alone

**Fig. 6:**
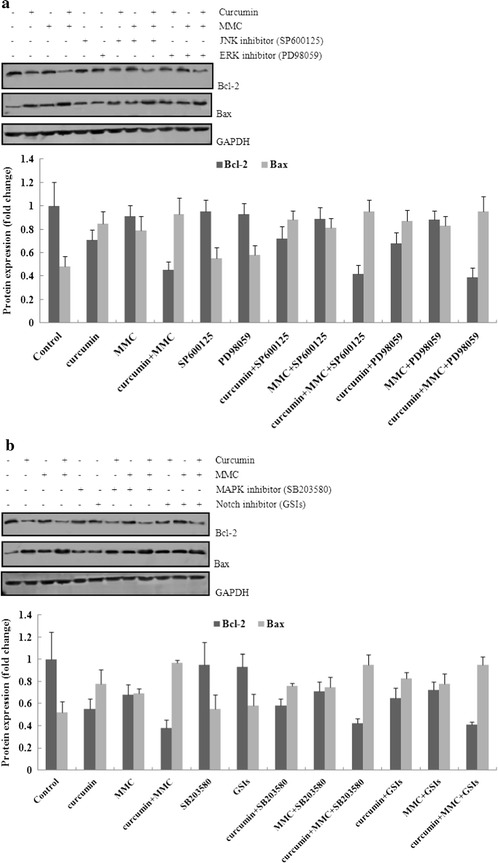
Effect of specific inhibitors of Wnt, Hedgehog, PI3k, NF-κB, JNK, ERK, MAPK, and Notch on Bcl-2 expression that was decreased by the combination treatment of curcumin and MMC. **a** SP600125 and PD98059; and **b** SB203580 and GSIs (10 μmol/L). Cells were then treated with 5 μmol/L curcumin and 0.1 μmol/L MMC alone or together for 7 days. Western blot analysis was then performed. The density ratio of proteins to GAPDH is shown as relative expression. Values are expressed as the mean ± SD (n = 3). Experiments were repeated with similar results. *P < 0.05 vs. specific inhibitors alone

**Fig. 7:**
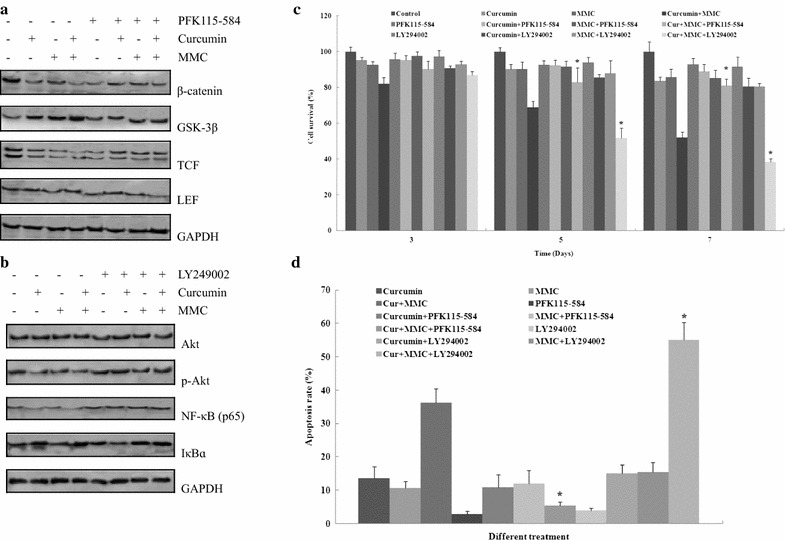
Effect of specific inhibitors of Wnt (PFK115-584) and PI3k (LY294002) on cell survival and apoptosis that was decreased and induced by the combination treatment of curcumin and MMC. MCF-7 BCSCs were pretreated for 2 h with or without 10 μmol/L PFK115-584 and LY294002 before cells were treated with 5 μmol/L curcumin and 0.1 μmol/L MMC alone or together. The associated proteins of the **a** Wnt and **b** PI3k signaling pathway were detected using western blot analysis. Cells were treated for 3, 5, and 7 days. MTT assay (**c**) and flow cytometry analysis (**D**) were performed as described in the methods section. Values are presented as the mean ± SD from three independent experiments. *P < 0.05 vs. the combination treatment of curcumin and MMC

In this study, PFK115-584, a Wnt-specific inhibitor, returned Bcl-2 expression induced by the combination treatment to the basal level. The PI3k inhibitor LY294002 further inhibited Bcl-2 expression following the combination treatment (Figs. 5a, b, 6). Moreover, β-catenin was disaggregated from the complex and translocated into the nucleus when the Wnt signaling was activated. Nuclear β-catenin forms a complex with TCF/LEF transcription factors to activate genes involved in tumorigenesis [50]. The PI3k signaling pathway is frequently activated in human cancers. AKT-mediated substrate phosphorylation regulates the transcription and translation of the genes required for cellular growth, metabolism, and survival [51]. We found that the Wnt and PI3K signaling pathways were regulated by combined treatment with curcumin and MMC (Fig. 7a, b). Cell survival and apoptosis were also affected by the combination treatment (Fig. 7c, d). Wnt’s bind to membrane receptor complexes comprised a frizzled G-protein-coupled receptor and a low-density lipoprotein receptor-related protein. The formation of this ligand-receptor complex initiates various signaling cascades, including the canonical β-catenin pathway and several noncanonical pathways [52]. However, the inhibition of the PI3K/Akt/mTOR pathway by small-molecule PI3K inhibitors primes cancer cells to mitochondrial apoptosis by tipping the balance toward proapoptotic Bcl-2 proteins, resulting in increased mitochondrial outer membrane permeabilization. Thus, mitochondrial apoptotic events play an important role in PI3k inhibitor-mediated sensitization for apoptosis. We conclude that the decrease of Bcl-2 following combined treatment with curcumin and MMC may be regulated by the Wnt and PI3k pathways.

## Conclusion

We characterized the effects and the mechanism of action of the combined treatment of curcumin and MMC in BCSCs. The results in this study suggest that the combined treatment (1) inhibits the self-renewal ability more significantly than MMC alone does; (2) induces apoptosis by regulating the imbalance of Bcl-2 family proteins; and (3) inhibits Bcl-2 expression via the Wnt and PI3k pathways. Therefore, the combined therapy of curcumin and MMC may be beneficial in the treatment of chemotherapy-resistant breast cancer.
